# Prognostic Implication of Human Papillomavirus Types and Species in Cervical Cancer Patients Undergoing Primary Treatment

**DOI:** 10.1371/journal.pone.0122557

**Published:** 2015-04-09

**Authors:** Yat Ming Lau, Tak Hong Cheung, Winnie Yeo, Frankie Mo, Mei Yung Yu, Kun Min Lee, Wendy C. S. Ho, Apple C. M. Yeung, Priscilla T. Y. Law, Paul K. S. Chan

**Affiliations:** 1 Department of Medicine and Geriatrics, United Christian Hospital, Hong Kong Special Administrative Region, People's Republic of China; 2 Department of Obstetrics and Gynaecology, Faculty of Medicine, The Chinese University of Hong Kong, Hong Kong Special Administrative Region, People's Republic of China; 3 State Key Laboratory in Oncology in South China, The Chinese University of Hong Kong, Hong Kong Special Administrative Region, People's Republic of China; 4 Sir YK Pao Center for Cancer, The Chinese University of Hong Kong, Hong Kong Special Administrative Region, People's Republic of China; 5 Department of Clinical Oncology, Faculty of Medicine, The Chinese University of Hong Kong, Hong Kong Special Administrative Region, People's Republic of China; 6 Hong Kong Cancer Institute and Prince of Wales Hospital, The Chinese University of Hong Kong, Hong Kong Special Administrative Region, People's Republic of China; 7 Department of Anatomical and Cellular Pathology, Faculty of Medicine, The Chinese University of Hong Kong, Hong Kong Special Administrative Region, People's Republic of China; 8 Department of Microbiology, Faculty of Medicine, Chinese University of Hong Kong, Hong Kong Special Administrative Region, People's Republic of China; IPO, Portuguese Institute of Oncology of Porto, PORTUGAL

## Abstract

High-risk human papillomavirus (HPV) types are associated with cervical cancer. It is well established that individual HPV types vary in oncogenicity, but current data on their prognostic implication remain controversial. We examined the association between HPV types/species and the survival of 236 Chinese women aged 26–87 (mean 54.4) years after receiving primary treatment for cervical cancer. Overall, 45.8% were of FIGO stage I, 41.9% stage II, and 12.3% stage III. The four most prevalent types found were HPV-16 (60.2%), HPV-18 (21.6%), HPV-52 (11.9%), and HPV-58 (9.3%). Overall, 19.5% of patients had multiple-type infections, 78.4% harboured one or more alpha-9 species, and 28.8% harboured one or more alpha-7 species. After a median follow-up of 8.0 years, 156 (66.1%) patients survived. The 3-year overall survival rate was 75.5%. Factors independently associated with a poorer 3-year overall survival were age >60 years, tumour size >4 cm, lymph node involvement and treatment with radiotherapy+/-chemotherapy. Univariate analysis showed HPV-16 single-type infection was associated with a marginally poorer disease-specific survival (71.6% vs. 87.0%, HR: 1.71, 95% CI = 1.01–2.90), whereas non-HPV-16 alpha-9 species was associated with a better disease-specific survival (90.0% vs. 76.2%, HR: 0.36, 95% CI = 0.16–0.79). However, on multivariate analysis, HPV infection status irrespective of different grouping methods, including individual types, species, single-type or co-infection, did not carry any significant prognostic significance. In conclusion, we did not observe any association between infection with a particular HPV type/species and survival. An HPV type-based stratification in treatment and follow-up plan could not be recommended.

## Introduction

Globally, cervical cancer is the fourth most common cancer in women with 528,000 new cases occurred in 2012 [1]. In Hong Kong, cervical cancer is the ninth commonest cancer in women with a crude incidence rate of 10.4 per 100,000 [2]. The aetiological association between infection with high-risk human papillomavirus (HPV) types and cervical cancer has been well established [3]. To date, over 150 HPV types have been identified which are classified into alpha-, beta-, gamma-, mu-, and nu-genera; under which they are further grouped into species and types [4]. HPV types associated with malignancy are referred as “high-risk”, which includes HPV-16, -18, -31, -33, -35, -39, -45, -51, -52, -56, -58, -59, -68, -73 and -82. “Low-risk” types including HPV-6, -11, -40, -42, -43, -44, -54, -61, -70, -72 and -81 are associated with benign anogenital lesions [5, 6]. Multiple HPV types are commonly found in cervical cancer specimens. However, the oncogenic implication of co-infection remains controversial [7–9]. The only tumor characteristics recognized to associate with HPV type is the higher prevalence of HPV 18 in cervical adenocarcinoma and adenosquamous carcinoma as compared to squamous cell carcinoma [10, 11].

High-risk HPV types demonstrate a biased distribution in cervical cancers as a result of their difference in oncogenicity and maybe, to a certain degree, their ethnogeographical distribution [12]. The eight most common HPV types found in cervical cancers worldwide in descending order are HPV-16, -18, -33, -45, -31, -58, -52, and -35. However, geographical variation in prevalence exists. For instance, HPV-52 and -58 are more prevalent in Asian cervical cancers as compared to other regions [13, 14].

The prognosis of cervical cancer is affected by staging, tumor size, parametrial and lymph node involvement. In early stage tumors, lymphovascular invasion and deep stromal invasion (>10 mm or >70% invasion) are associated with poor prognosis. Previous studies suggest a poorer outcome for adenocarcinoma [15–17]. Although the oncogenic potential of HPV types has been well established, their prognostic significance remains controversial. Several studies found that HPV-18 conferred poor prognosis in early stage cervical cancers [11, 18–20]. De Cremoux et al. reported that high-risk HPV types were associated with reduced disease-free survival as compared to intermediate-risk types [21]. However, the study from Tong et al. did not observe any difference in overall survival between high-risk and intermediate-risk types [22]. Lai et al. reported a better 5-year survival rate for HPV-58 and the related types (HPV-52/-33) as compared to HPV-16/-18 and the related types (HPV-31/-68) [23]. Huang et al. reported that HPV-31 and the related types (HPV-33, -35 and -67) could predict a better survival [24]. Wang et al. compared patients infected with alpha-7 species only, alpha-9 species only, and co-infection with alpha-7 and alpha-9 species; and concluded that these groups conferred poorer, better and intermediate survival outcome, respectively [25]. However, negative observations have been reported by others [22, 26–28].

Analysis on the prognosis of different HPV types is complicated by the fact that co-infection with multiple types is common and little is known about the interaction between the co-infecting types. The much skewed prevalence of certain HPV types, e.g. HPV52 and HPV58, also poses challenge. This study evaluated the prognostic significance of HPV types with a focus on co-infection and those types, HPV-52 and -58, commonly found in our locality.

## Method

### Study population

Patients who had received operation for cervical cancer at the Prince of Wales Hospital between 1997 and 2009 were recruited with written informed consent. Medical records were reviewed in 2012 via the electronic Clinical Management System to determine outcomes. Pathological and radiological reports were retrieved to ascertain findings of clinical notes. Pathological features including tumor stage, size, histological type and grade, parametrial, vaginal and lymph node involvement were recorded at the time of diagnosis. The date of primary treatment defined the commencement of survival count. The date of diagnosis of relapse by imaging, biopsy or clinical examination defined the date of relapse. Disease-specific death was defined as death resulting from cervical cancer and related complications or treatment toxicity. The study was approved by the local institutional ethics committee, The Joint Chinese University of Hong Kong—New Territories East Cluster Clinical Research Ethics Committee, with approval number CRE-2008.200.

### HPV detection and typing

Fresh frozen tumor tissues were collected by cervical biopsy or during operation. The methods of HPV DNA detection and typing were described previously [14, 26]. Briefly, HPV DNA was detected by polymerase chain reaction (PCR) using consensus primers, PGMY09/11. HPV-positive specimens were typed by the Linear Array HPV Genotyping Test (Roche, Molecular Systems, Inc., CA). As according to manufacturer’s instructions, all samples showing a positive band that may indicate the presence of HPV52 were confirmed by HPV52-specific PCR using primers M13-HPV52-pE7-F2: 5’-TGT AAA ACG ACG GCC AGT GGA GGA TAC AGA TGG TGT GG-3’; M13-HPV 52-pE7-R: 5’-CAG GAA ACA GCT ATG ACC ATG AAT GCA GCC GTA GTG-3’. In view of the low positive rate of HPV-45 among adenocarcinoma cases, all adenocarcinoma specimens were tested, in addition, by an HPV-45-specific PCR targeting the E7 gene using primers: 5’-CCR RGM AAC ACT GCA AGA AAT T-3’ and 5’-CGC GCT GGT AGT TGT GCA TGA C-3’.

HPV types are grouped according to the species classification adopted by the International Committee on the Taxonomy of Viruses [29]. Alpha-9 species includes HPV-16, -31, -33, -35, -52, -58 and -67; and alpha-7 species includes HPV-18, -39, -45, -59, -68, -70 and -85.

### Treatment and follow-up

Patients were treated according to the departmental protocol which was constantly updated according to contemporary guidelines. Treatment plans were decided in Gynaecological Tumor Board meetings consisting of gynaeoncologists, medical and radiation oncologists as well as pathologists. The International Federation of Gynecology and Obstetrics (FIGO) staging system was used. In general, adjuvant treatment with concurrent chemoradiation (CCRT) are given for resected stage I–IIA disease with poor prognostic factors including pelvic lymph node involvement, parametrial disease or positive resection margin. Post-operative adjuvant radiotherapy (RT) alone was used for patients who were unfit for or declined CCRT.

Primary CCRT was the treatment for stage IB / bulky stage IIA (> 4 cm) / stage IIB- IVA disease or any stage with pelvic lymph node involvement. Primary RT alone was an alternative to surgery for patients with stage IA or non-bulky stage IIA disease who were unfit for surgery. Primary RT alone was an alternative treatment for patients with stage IB / bulky IIA (> 4cm) / stage IIB to IVA disease or for those with pelvic lymph node involvement who were unfit for or declined CCRT.

For patients treated with CCRT, weekly cisplatin 40 mg / m^2^ for 6 cycles were given concurrently with external radiotherapy as radiosensitiser, starting 1 day before Day-1 radiotherapy. For radiotherapy, patient underwent a course of external beam irradiation combined with intracavitary brachytherapy. External beam irradiation to whole pelvis was given at 1.8 Gy per fraction, 5 times per week with a total dose of 45–54 Gy. Intracavitary brachytherapy (ICB) was given weekly for 3 doses using the high-dose rate (HDR) afterloading technique, delivering 7 Gy per fraction to Point A. Additional parametrial boost (usually 5.4–9.0 Gy over 3–5 fractions) was given with dose calculated according to the stage and normal tissue tolerance. Abdominal para-aortic region were irradiated if para-aortic lymph node involvement was suspected.

Patients had the first follow-up visit at 6 weeks post-treatment, and then every 3 months in the first year, every 4 months in the second year, every 6 months in the third to fifth year, and yearly from the sixth year onwards. Physical examinations including per-vaginal and per-rectal examination were performed at each follow-up. Pap smear and imaging were arranged as clinically indicated.

### Statistical analysis

Statistical analyses were performed using the Statistical Analysis System (SAS) version 8.2. Kaplan-Meier method was used for survival analysis. Survival curves were compared by the log-rank test. Hazard ratio (HR) was determined by Cox regression in univariate analysis. Variables showing significant associations in univariate analysis were tested in multivariate model using Cox regression with stepwise selection. All p-values were two-sided. A p-value of < 0.05 was considered statistically significant.

## Results

A total of 271 patients with newly diagnosed FIGO stage I-IV diseases were recruited, of whom 35 were excluded from survival analysis because of stage IV disease in 21 cases, stage I-III disease but refused treatment in 10 cases, and incomplete medical record in 4 cases. Hence, a total of 236 patients were included in this study. The clinical information database is shown in S1 Table.

**Fig 1:**
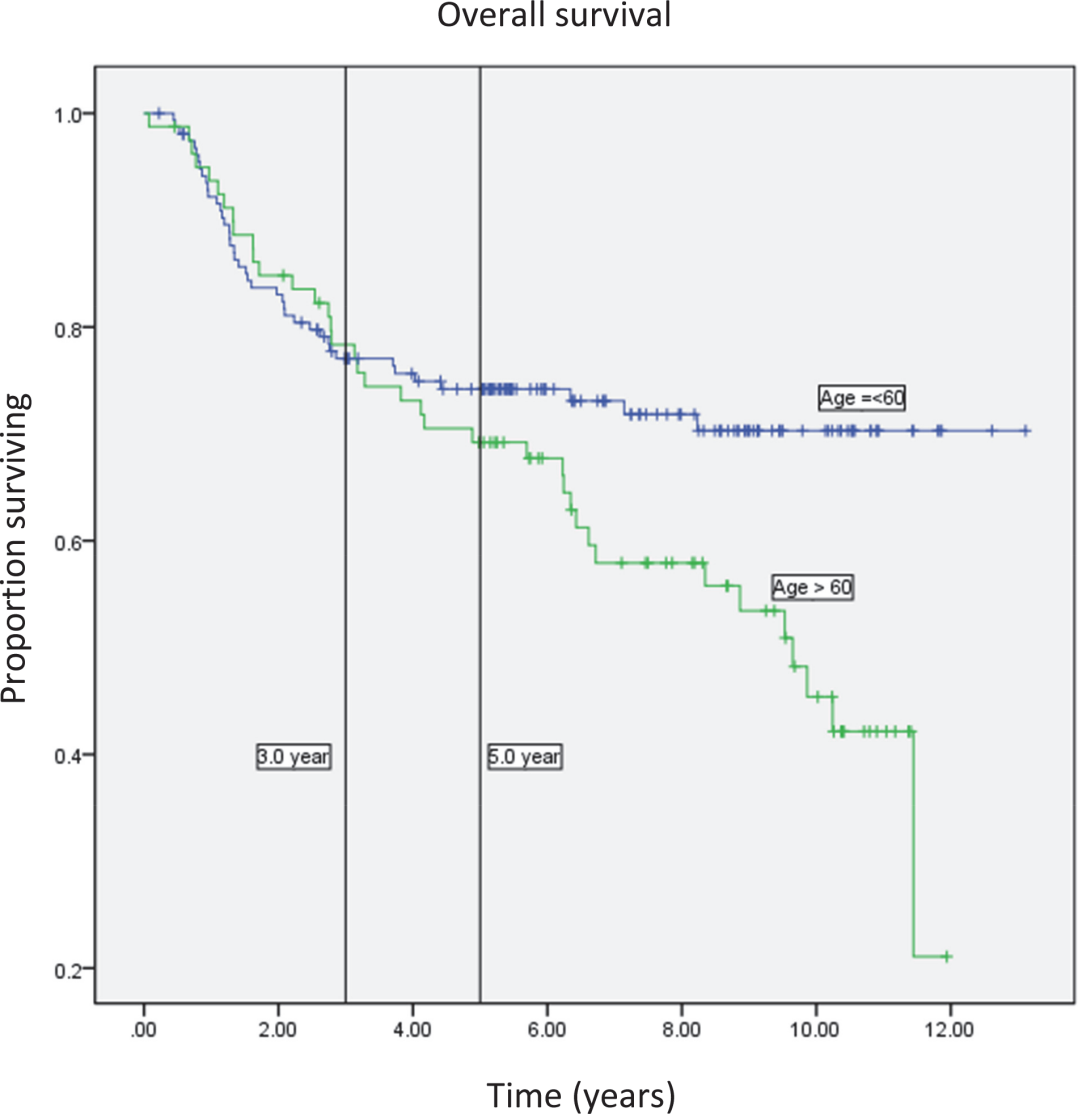
Kaplan-Meier overall survival curve of patients aged 60 years and below compared to greater than 60 years.

The 236 study subjects aged 26–87 (mean 54.4, 95% confidence interval [CI] = 52.7–56.1) years, and with a median follow-up time of 8.0 (95% CI = 7.2–8.7) years. Sixty-one patients had recurrence, with local relapse in 6 cases, pelvic relapse in 7 cases, and distant relapse in 45 cases. Information on the site of relapse was not available in 3 cases. The 3-year overall survival, disease-free survival, and disease-specific survival was 75.5%, 78.5% and 79.9%, respectively. There were 80 deaths at the time of study analysis on 2 April 2012, of whom 57 were related to cervical cancer and 21 were due to other causes. The cause of death was missing in 2 cases, which were censored at the date of death in the Kaplan-Meier analysis. Though age > 60 years had marginally better 3-year overall survival (78.3% vs 77.1%, p = 0.02), advanced age had poorer overall survival in long term (Fig 1). Other characteristics of study patients are shown in Table 1.

**Table 1:** Clinicopathologic characteristics of study patients.

Characteristics	No. of patients (%), N = 236
Age (years)	
≤60	156 (66.1)
>60	80 (33.9)
Smoking status	
Never smoker	151 (64.0)
Ex-smoker	7 (3.0)
Chronic smoker	14 (5.9)
Unknown	64 (27.1)
FIGO stage	
Stage I (total)	108 (45.8)
IA1	1 (0.4)
IA2	1 (0.4)
IB1	91 (38.6)
IB2	15 (6.4)
Stage II (total)	99 (41.9)
IIA1	19 (8.0)
IIA2	5 (2.1)
IIB	75 (31.8)
Stage III (total)	29 (12.3)
IIIA	8 (3.4)
IIIB	21 (8.9)
Histological type	
Squamous cell carcinoma	186 (78.8)
Adenocarcinoma	41 (17.4)
Adenosquamous cell carcinoma	9 (3.8)
Grade of differentiation	
Well	4 (1.7)
Moderate	69 (29.4)
Poor	44 (18.6)
Not reported	119 (50.4)
Tumor size	
<4cm	136 (57.6)
≥4cm	100 (42.4)
Parametrial involvement	
Yes	105 (44.5)
No	131 (55.5)
Vaginal involvement	
Yes	118 (50.0)
No	118 (50.0)
Regional lymph node metastasis	
Yes	59 (25.0)
No	177 (75.0)
Primary treatment	
Surgery +/- adjuvant	74 (31.4)
Radiotherapy +/- chemotherapy	160 (67.8)
Palliative radiotherapy or chemotherapy	2 (0.8)

High-risk HPV types were found in 98.3% of tumors, whereas 4 (1.7%) tumors contained exclusively low-risk types including one case of HPV-26, one case of HPV-70, and two cases of HPV-53. The 4 most common HPV types detected were HPV-16 (60.2%), HPV-18 (21.6%), HPV-52 (11.9%) and HPV-58 (9.3%) (Table 2). Altogether, 185 (78.4%) specimens harbored HPV of the alpha-9 species, while 68 (28.8%) harbored alpha-7 species (Table 3). Overall, 190 (80.5%) specimens contained single-type infection, while 46 (19.5%) harbored multiple HPV types with 30 (12.7%) specimens contained 2 types, 10 (4.2%) specimens contained 3 types, 2 (0.8%) specimens contained 4 types, and 4 (1.7%) specimens contained 5 types. Co-infection rate of HPV-16, HPV-18, HPV-52, HPV-58 containing tumors were 24.6%, 41.0%, 60.7% and 54.5%, respectively.

**Table 2:** Prevalence of human papillomavirus types among study patients.

Characteristics	No. of patients (%), N = 236
HPV infection	
Single-type	190 (80.5)
Multiple-type	46 (19.5)
HPV type detected	
HPV-16	142 (60.2)
HPV-18	51 (21.6)
HPV-52	28 (11.9)
HPV-58	22 (9.3)
HPV-31	9 (3.8)
HPV-33	8 (3.4)
HPV-45	7 (3.0)
HPV-35	5 (2.1)
HPV-68	4 (1.7)
HPV-67	3 (1.3)
HPV-53	2 (0.8)
HPV-62	2 (0.8)
HPV-39	2 (0.8)
HPV-11	1 (0.4)
HPV-26	1 (0.4)
HPV-51	1 (0.4)
HPV-54	1 (0.4)
HPV-56	1 (0.4)
HPV-61	1 (0.4)
HPV-69	1 (0.4)
HPV-70	1 (0.4)
HPV-73	1 (0.4)
HPV-82	1 (0.4)
HPV-108	1 (0.4)

**Table 3:** Prevalence of common human papillomavirus types and alpha-7 and -9 species among study patients

HPV type / species	No. of patients (%), N = 236
HPV 16 +ve	142 (60.2)
HPV 16 +ve, single infection	107 (45.3)
HPV 16 +ve, co-infection	35 (14.8)
HPV 16 −ve	94 (39.8)
HPV 18 +ve	51 (21.6)
HPV 18 +ve, single infection	30 (12.7)
HPV 18 +ve, co-infection	21 (8.9)
HPV 18 −ve	185 (78.4)
HPV 52 +ve	28 (11.9)
HPV 52 +ve, single infection	11 (4.7)
HPV 52 +ve, co-infection	17 (7.2)
HPV 52 −ve	208 (88.1)
HPV 58 +ve	22 (9.3)
HPV 58 +ve, single infection	10 (4.2)
HPV 58 +ve, co-infection	12 (5.1)
HPV 58 −ve	214 (90.7)
Alpha-9 species	185 (78.4)
Alpha-7 species	68 (28.8)
Alpha-9 species (not including HPV16):	
HPV-31/-33/-35/-52/-58/-67 +ve	65 (27.5)
Alpha-7 species (not including HPV18):	
HPV-39/-45/-59/-68/-70/-85 +ve	22 (9.3)

### Univariate analysis

Six clinicopathological features, including FIGO stage, tumor size > 4 cm, parametrial involvement, vaginal involvement, lymph node involvement and primary treatment modality, were significantly associated with all survival parameters including 3-year overall survival, 3-year disease-specific survival and 3-year disease-free survival by univariate analysis (Table 4).

**Table 4:** Univariate analysis on association between clinicopathologic characteristics and survival of patients with cervical cancer after primary treatment.

Characteristics	No. of patients (N = 236)	3-year overall survival				3-year disease-specific survival				3-year disease-free survival			
		%	p-value	HR	95% CI for HR	%	p-value	HR	95% CI for HR	%	p-value	HR	95% CI for HR
Age (years)			**0.02**	**1.65**	**1.06–2.56**		0.63	0.87	0.50–1.52		0.32	0.76	0.44–1.32
≤60	156	**77.1**	** **	** **	** **	78.3				76.9			
>60	80	**78.3**	** **	** **	** **	83				81.6			
Smoking			0.15	0.94	0.88–1.00		0.48	0.96	0.90–1.03		0.52	0.98	0.92–1.05
Never smoker	151	76.5				78.9				78			
Ex-smoker	7	71.4				71.4				71.4			
Chronic smoker	14	57.1				77.1				62.9			
unknown	64	83.6				83.6				84			
FIGO stage (original stage)			**<0.01**		** **		**<0.01**				**<0.01**		
StageI (total)	108	**84.6**	** **	1	** **	86.6	** **	1		85.6	** **	1	
IA1	1	**100**	** **		** **	100	** **			100	** **		
IA2	1	**100**	** **		** **	100	** **			100	** **		
IB1	91	**89.7**	** **		** **	92	** **			87.6	** **		
IB2	15	**50.3**	** **		** **	50.3	** **			67.4	** **		
Stage II (total)	99	**75.7**	** **	**1.74**	**1.05–2.89**	79.3	** **	1.59	0.87–2.91	77.1	** **	1.69	0.95–3.01
IIA1	19	**73.7**	** **		** **	77.8	** **			77.8	** **		
IIA2	5	**80**	** **		** **	80	** **			80	** **		
IIB	75	**76**	** **	** **	** **	79.6	** **	** **		76.7	** **		** **
Stage III (total)	29	**56.7**	** **	**3.74**	**1.76–6.10**	**56.7**	** **	**3.29**	**1.61–6.72**	**56.2**	** **	**3.26**	**1.61–6.60**
IIIA	8	**87.5**	** **			**87.5**	** **			**75**	** **		
IIIB	21	**45**	** **			**45**	** **			**48.7**	** **		
Histological type			0.37	1.00	0.66–1.50		0.4	1.12	0.75–1.86		0.06	1.3	0.86–1.97
Squamous cell carcinoma	186	77.1				79				80.1			
Adenocarcinoma	41	76.8				81.9				68.3			
Adenosquamous cell carcinoma	9	88.9				88.9				88.9			
Grade of differentiation			0.11	1.83	1.03–3.24		0.12	1.88	1.02–3.48		0.06	2.16	1.14–4.09
Well	4	100				100				100			
Moderate	69	80.8				82.4				81.9			
Poor	44	63.3				65.6				60.3			
Tumor size		** **	**<0.01**	**2.27**	**1.46–3.55**	** **	**<0.01**	**3.56**	**2.04–6.23**	** **	**<0.01**	**2.8**	**1.67–4.72**
<4cm	136	**85.7**	** **	** **	** **	**88.6**	** **	** **	** **	**87.3**	** **	** **	** **
≥4cm	100	**66.3**	** **	** **	** **	**68**	** **	** **	** **	**65.8**	** **	** **	** **
Parametrial involvement		** **	**0.01**	**1.85**	**1.19–2.89**	** **	**<0.01**	**2.15**	**1.26–3.66**	** **	**0.01**	**1.95**	**1.17–3.26**
Yes	105	**71.7**	** **	** **	** **	**73.6**	** **	** **	** **	**70.1**	** **	** **	** **
No	131	**82.7**	** **	** **	** **	**85**	** **	** **	** **	**85.1**	** **	** **	** **
Vaginal involvement		** **	**<0.01**	**2.46**	**1.54–3.96**	** **	**<0.01**	**2.66**	**1.51–4.69**	** **	**<0.01**	**2.28**	**1.34–3.90**
Yes	118	**68.3**	** **	** **	** **	**71.1**	** **	** **	** **	**69.9**	** **	** **	** **
No	118	**86.8**	** **	** **	** **	**88.6**	** **	** **	** **	**86.9**	** **	** **	** **
Lymph node metastasis		** **	**<0.01**	**3.44**	**2.20–5.39**	** **	**<0.01**	**5.32**	**3.15–9.01**	** **	**<0.01**	**4.63**	**2.78–7.68**
Yes	59	**52.2**	** **	** **	** **	**52.2**	** **	** **	** **	**51.4**	** **	** **	** **
No	177	**85.7**	** **	** **	** **	**90**	** **	** **	** **	**87.2**	** **	** **	** **
Primary treatment			**<0.01**	** **	** **	** **	**<0.01**			** **	**<0.01**	** **	** **
Surgery +/- adjuvant	74	**92.9**	** **	**1**	** **	**94.3**	** **	**1**	** **	**91.6**	** **	**1**	** **
Radiotherapy +/- chemotherapy	160	**70.4**	** **	**5.19**	**2.39–11.28**	**73.2**	** **	**4.67**	**1.94–11.21**	**72.2**	** **	**3.42**	**1.62–7.20**
Palliative radiotherapy or chemotherapy	2	**0**	** **	**10.35**	**1.27–84.34**	**0**	** **	**4.03**	**1.63–9.99**	**0**	** **	**7.82**	**0.98–62.66**

HR, hazard ratio; CI, confidential interval; statistically significant associations are bolded.

Tumors harbouring single-type HPV infection exhibited a poorer 3-year disease-free survival compared to those with multiple types (76.1% vs. 88.8%), but the difference was only at the margin of statistical significance (p = 0.05, HR = 0.44, 95% CI = 0.19–1.02) (Table 5). HPV-16 (single infection) was associated with a poorer 3-year disease-specific survival (71.6% vs. 87.0%, p = 0.04, HR = 1.71, 95% CI = 1.01–2.90). The presence of non-HPV-16 alpha-9 species was associated with a better 3-year disease-specific survival (90.0% vs. 76.2%, p = 0.01, HR = 0.36, 95% CI = 0.16–0.79) and 3-year disease-free survival (87.1% vs. 75.3%, p = 0.02, HR = 0.45, 95% CI 0.22–0.91). HPV-18 whether alone or co-infected with other types did not confer a significant difference in survival.

**Table 5:** Univariate analysis on association between HPV types and survival of patients with cervical cancer after primary treatment.

HPV infection status	No. of patients (N = 236)	3-year overall survival				3-year disease-specific survival				3-year disease-free survival			
		%	p-value	HR	95% CI for HR	%	p-value	HR	95% CI for HR	%	p-value	HR	95% CI for HR
Single-type infection	190	74.9	0.13	0.6	0.31–1.17	77.8	0.15	0.56	0.25–1.24	76.1	0.05	0.44	0.19–1.02
Multiple-type infection	46	88.4				88.4				88.8			
HPV-16 +ve, regardless of single or co-infection	142	74.7	0.85	0.96	0.61–1.50	74.7	0.08	1.65	0.93–2.91	76.7	0.48	1.21	0.72–2.04
HPV-16 −ve	94	81.7				87.9				81.2			
HPV-18 +ve, regardless of single or co-infection	51	84	0.86	0.95	0.56–1.63	88	0.82	0.93	0.49–1.76	79.7	0.86	1.05	0.58–1.91
HPV-18 −ve	185	75.7				77.7				78.2			
HPV-52 +ve, regardless of single or co-infection	28	85.6	0.88	0.95	0.47–1.90	88.7	0.22	0.54	0.19–1.48	85.3	0.18	0.51	0.18–1.40
HPV-52 −ve	208	76.4				78.7				77.6			
HPV-58 +ve, regardless of single or co-infection	22	80.1	0.49	1.28	0.64–2.56	84.1	0.29	0.54	0.17–1.72	80.3	0.84	0.91	0.36–2.27
HPV-58-ve	214	77.2				79.4				78.3			
HPV-16 +ve, single infection	107	71.6	0.97	0.97	0.61–1.56	71.6	0.12	1.794	1.00–3.22	73.2	0.17	1.39	0.82–2.38
HPV-16 +ve, co-infection	35	84.4		0.91	0.45–1.86	84.4		1.189	0.49–2.69	88.1		0.64	0.24–1.68
HPV-16 −ve	94	81.7		1	-	87.9		1	-	81.2		1	-
HPV-16 +ve, single infection	107	71.6	0.98	0.99	0.64–1.55	**71.6**	**0.04**	**1.71**	**1.01–2.90**	73.2	0.09	1.54	0.93–2.56
HPV-16 +ve, co-infection & HPV-16 −ve	129	82.4				**87**				83			
HPV-16 +ve, single infection	107	71.6	0.74	0.89	0.44–1.80	71.6	0.3	0.652	0.29–1.47	73.2	0.07	0.44	0.17–1.11
HPV-16 +ve, co-infection	35	84.4				84.4				88.1			
HPV-18 +ve, single infection	30	80	0.54	1.18	0.64–2.19	86.5	0.52	1.189	0.58–2.43	73.2	0.29	1.42	0.74–2.74
HPV-18 +ve, co-infection	21	90		0.65	0.26–1.63	90		0.563	0.18–1.81	90.2		0.54	0.17–1.74
HPV-18 −ve	185	75.7		1	-	77.7		1	-	78.2		1	-
HPV-18 +ve, single infection	30	80	0.52	1.23	0.66–2.27	86.5	0.54	1.247	0.61–2.54	73.2	0.23	1.49	0.78–2.86
HPV-18 +ve, co-infection & HPV-18 −ve	206	77.1				78.9				79.4			
HPV-18 +ve, single infection	30	80	0.26	0.55	0.19–1.57	96.5	0.23	0.455	0.12–1.68	73.2	0.12	0.38	0.11–1.35
HPV-18 +ve, co-infection	21	90				90				90.2			
HPV-52 +ve, single infection	11	71.6	0.46	1.49	0.60–3.69	78.8	0.43	0.76	0.19–3.13	70.7	0.17	1.17	0.37–3.74
HPV-52 +ve, co-infection	17	94.1		0.65	0.24–1.79	94.1		0.41	0.10–1.70	94.1		0.19	0.03–1.36
HPV-52 −ve	208	76.4		1	-	78.7		1	-	77.6		1	-
HPV-52 +ve, single infection	11	71.6	0.36	1.53	0.62–3.79	78.8	0.76	0.80	0.20–3.29	70.7	0.70	1.26	0.39–4.01
HPV-52 +ve, co-infection & HPV-52 −ve	225	77.7				79.9				78.9			
HPV-52 +ve, single-infection	11	71.6	0.18	0.42	0.11–1.56	78.8	0.5	0.51	0.07–3.67	70.7	0.1	0.19	0.02–1.79
HPV-52 +ve, co-infection	17	94.1				94.1				94.1			
HPV-58 +ve, single infection	10	80	0.16	1.95	0.90–4.25	88.9	0.53	0.37	0.05–2.70	78.8	0.87	1.12	0.35–3.57
HPV-58 +ve, co-infection	12	78.8		0.58	0.14–2.36	78.8		0.69	0.17–2.82	82.5		0.71	0.17–2.92
HPV-58 −ve	214	77.2		1	-	79.4		1	-	78.3		1	-
HPV-58 +ve, single infection	10	80	0.08	1.99	0.92–4.33	88.9	0.08	1.99	0.92–4.33	78.8	0.83	1.13	0.36–3.62
HPV-58 +ve, co-infection & HPV-58 −ve	226	77.4				79.5				78.5			
HPV-58 +ve, single infection	10	80	0.2	0.36	0.07–1.82	88.9	0.65	1.74	0.16–19.23	78.8	0.92	0.9	0.13–6.43
HPV-58 +ve, co-infection	12	78.8				78.8				82.5			
Alpha-9 species +ve^1^	185	77.3	0.68	0.89	0.53–1.52	78.7	0.58	0.84	0.46–1.54	79.3	0.34	0.76	0.43–1.34
Alpha-9 species-ve	51	78				83.9				75.6			
Alpha-7 species +ve^2^	68	83	0.56	0.86	0.52–1.41	86.1	0.71	0.9	0.50–1.61	81.2	0.68	0.89	0.50–1.57
Alpha-7 species-ve	168	75.4				77.5				77.4			
Alpha-9 species (excluding HPV-16) +ve	65	85.6	0.89	0.97	0.59–1.59	**90**	**0.01**	**0.36**	**0.16–0.79**	**87.1**	**0.02**	**0.45**	**0.22–0.91**
Alpha-9 species (excluding HPV-16)-ve	171	74.5				**76.2**				**75.3**			
Alpha-7 species (excluding HPV-18) +ve	22	79.8	0.6	0.79	0.32–1.94	80	0.9	1.06	0.42–2.65	85.2	0.52	0.74	0.30–1.84
Alpha-7 species (excluding HPV-18)-ve	214	77.3				79.9				77.9			

^1^ Alpha-9 species includes HPV-16, -31, -33, -35, -52, -58, and -67

^2^ Alpha-7 species includes HPV-18, -39, -45, -59, -68, -70, and -85

HR, hazard ratio; CI, confidence interval; statistically significant associations are bolded

### Multivariate analysis

Clinicopathological characteristics showing a significant association with any survival parameter in univariate analysis were tested in multivariate model by cox regression with stepwise selection. Furthermore, selected HPV infection statuses were added individually to the multivariate model, including: (i) HPV-16, -18, -52, -58 infection status classified with respect to single-type or co-infection; (ii) grouped into alpha-7 and -9 species.

Four clinicopathological characteristics were found to be independent prognostic factors for a poorer 3-year overall survival, including (i) lymph node involvement (HR = 4.17, 95% CI = 2.51–6.94); (ii) tumor size > 4 cm (HR = 1.83, 95% CI = 1.11–3.02); (iii) age > 60 years (HR = 2.53, 95% CI = 1.49–4.29); and (iv) radiotherapy/chemoradiation vs. surgery+/- adjuvant (HR = 2.89, 95% CI = 1.30–6.45). Furthermore, lymph node involvement (HR = 4.19, 95% CI = 2.45–7.16), tumor size (HR = 2.03, 95% CI = 1.12–3.68), and treatment modality (HR = 2.77, 95% CI 1.15–6.69) were independent prognostic factors for disease-specific survival. Whereas, lymph node involvement (HR = 4.23, 95% CI = 2.54–7.05) and treatment modality (HR = 2.87, 95% CI = 1.45–5.71) were independent prognostic factors for disease-free survival. However, infection with different HPV types, regardless of grouping methods, was not predictive of overall survival, disease-specific survival or disease-free survival in the multivariate model.

## Discussion

The distribution of HPV types observed in this study is consistent with previous reports that higher prevalence of HPV-52 and HPV-58 are found in Asia and parts of China [13, 14, 30, 31]. Of note, 21.2% of our cases were adenocarcinoma and adenosquamous cell carcinoma in which HPV18 constituted a large portion. We could not observe any independent association between the most prevalent HPV types (HPV-16, -18, -52 and -58) with post-treatment survival among patients with cervical cancer. Although, univariate analysis suggested that HPV-16 might predict a poorer survival, this association could not be confirmed in multivariate model. Similarly, non-HPV-16 alpha-9 species as a group was associated with a better 3-year disease-specific survival in univariate analysis, but not in multivariate analysis. Whereas, for other common HPV types (HPV-18, -52, and -58), no significant association could be observed even in univariate analysis. Our findings are in line with the negative observations reported by Pilch et al. [26], Zampronha Rde et al. [27], and Ikenberg et al. [28], but are in contrary to others. Wang et al. analyzed 1010 cervical cancer patients underwent primary radiotherapy, and demonstrated a better survival for alpha-9 species compared to alpha-7 species, while co-infections of alpha-9 and alpha-7 species were found to have an intermediate survival [25]. Although we observed a better survival for alpha-9 species, the association could not be confirmed in multivariate model. Lai et al. studied 1067 patients with early stage cervical cancer who underwent primary surgery, and showed that HPV-18 positivity was an independent predictor of relapse [11]. Burger et al. examined 171 stage I and II patients undergoing primary surgery, and reported that HPV-18 was a poor prognostic factor [18]. In the current study, we observed a slightly better prognosis for HPV-18, but the differences were not statistically significant even in univariate analysis.

We tried to analyze the prognostic implication of multiple-type infections as a whole, as well as subgroups according to the presence of the four most prevalent types HPV-16, -18, -52 and -58. As in line with previous studies, no significant independent association could be demonstrated [11, 24, 25].

To the best of our knowledge, this is the first study on association between HPV infection and survival in Hong Kong where HPV-52 and HPV-58 are more prevalent. We used a sensitive method capable of detecting a wide range of HPV types as well as co-infections, which allows an in-depth analysis on different infection statuses. Furthermore, the sufficient duration of follow-up provides substantial strength to the study. Nevertheless, we have limitations. For instance, the number of study subjects was relatively small, especially for non-HPV-16 types, and the retrospective nature in data collection might pose biases. The reason for diverse observations among studies is not clear. Most studies were only able to compare a common type, often HPV-16/-18, with a heterogeneous group of different HPV types, in which the composition varies geographically. Another unexplored issue is whether the association with poorer survival reported in some localities could be due to the circulation of variants with higher oncogenicity and thus conferring a higher chance of recurrence. In addition, host genetic factors such as HLA polymorphisms which vary with ethical groups, might influence susceptibility to cervical cancer as well as the clinical outcomes after infection with certain HPV types [32–36].

In conclusion, we did not observe any prognostic value of HPV type for Hong Kong Chinese patients receiving primary treatment for cervical cancer. Risk stratified treatment or follow-up strategy based on HPV type could not be recommended.

## Supporting Information

S1 Table Clinical information database of study subjects. (XLSX)
